# *In vitro* antimicrobial activity of plants used in traditional medicine in Gurage and Silti Zones, south central Ethiopia

**DOI:** 10.1186/s12906-015-0822-1

**Published:** 2015-08-18

**Authors:** Alemtshay Teka, Johana Rondevaldova, Zemede Asfaw, Sebsebe Demissew, Patrick Van Damme, Ladislav Kokoska, Wouter Vanhove

**Affiliations:** Laboratory for Tropical and Subtropical Agriculture and Ethnobotany, Department of Plant Production, Faculty of Bio-Science Engineering, Ghent University, Coupure links, 653-9000 Ghent, Belgium; Department of Plant Biology and Biodiversity Management, College of Natural Sciences, Addis Ababa University, P.O. Box 3434, Addis Ababa, Ethiopia; Department of Crop Sciences and Agroforestry, Faculty of Tropical AgriSciences, Czech University of Life Sciences Prague, Kamycka 129, 165 21 Prague 6-Suchdol, Czech Republic

**Keywords:** Antibiotic-resistance, Anti-staphylococcal, Ethnomedicine, Ethnopharmacology, *Guizotia schimperi*

## Abstract

**Background:**

To overcome the escalating problems associated with infectious diseases and drug resistance, discovery of new antimicrobials is crucial. The present study aimed to carry out *in vitro* antimicrobial analysis of 15 medicinal plant species selected according to their traditional medicinal uses in Gurage and Silti Zones, south central Ethiopia.

**Methods:**

Ethanol extracts of various plant parts were investigated for their antimicrobial activity against 20 bacterial and one yeast strains. The minimum inhibitory concentration (MIC) was determined by broth microdilution method.

**Results:**

*Asparagus africanus, Guizotia schimperi*, *Lippia adoensis* var. *adoensis* and *Premna schimperi* were active against *Candida albicans, Enterococcus faecalis* and *Staphylococcus aureus* at a concentration of 512 μg/ml or lower. Strong antibacterial activity (MIC ≥ 128 μg/ml) was observed for *G. schimperi* extract against 17 resistant and sensitive *Staphylococcus* strains, at a concentration comparable to standard antibiotics. Moreover, this extract showed higher antibacterial activity for the test against *S. aureus* ATCC 33591, ATCC 33592, SA3 and SA5 strains (128–256 μg/ml) than oxacillin (512 μg/ml).

**Conclusions:**

The study revealed *in vitro* antibacterial activity of plants used in folk medicine in south central Ethiopia. The usefulness of these plants, in particular of *G. schimperi,* should be confirmed through further phytochemical and toxicity analyses.

## Background

Infectious diseases are an important cause of mortality and morbidity, in all regions of the world. The increasing emergence of antimicrobial resistance worsens the impact [1, 2]. It has been shown that risk of negative clinical consequences, mortality, and high treatment costs with drug-resistant bacteria is generally higher compared to patients infected with the same non-resistant bacteria [3]. Increased prevalence of resistant bacteria, together with lack and high cost of new generation drugs has escalated infection-related morbidity and mortality particularly in developing countries like Ethiopia [1, 4].

Numerous biochemical compounds obtained from medicinal plants possess important antimicrobial properties [5]. Application of these compounds is preferred over synthetic drugs as they have long been used in traditional medicine and are considered safe to humans [6]. New and effective antimicrobials identified from plants can consequently be considered in development of new drugs to combat problems associated with drug resistance [7]. Using effective plant extracts to control human diseases has the additional advantage of low production cost, minimal environmental damage and higher accessibility to rural communities [4, 8, 9]. Hence, medicinal plants are expected to be the future alternative source of new antimicrobial products [5, 10].

Treatment of infections with plant-derived compounds is an age-old practice that is globally employed, especially in developing countries [11, 12]. This point applies particularly to Ethiopia, where dramatic geographic, climatic and cultural diversity contribute to a wide range of traditional herbal knowledge and practices by the people [13, 14]. Numerous *in vitro* studies have been undertaken, and have revealed the antimicrobial potential of herbal medicines traditionally used in various regions of Ethiopia [11, 15–19]. However, many Ethiopian medicinal plants still await scientific validation of their anti-infective properties. The aim of this study was to assess *in vitro* antimicrobial activity of medicinal plant species selected based on their traditional medicinal uses for infectious diseases treatment in local community of Gurage and Silti Zones, south central Ethiopia. This analysis may also offer a source of information to identify effective medicinal plants against staphylococcal infections and facilitate selection of plants for further phytochemical investigation.

## Methods

### Selection of plants

Medicinal plants were collected from Gurage and Silti Zones, south central Ethiopia. Specimens were collected, pressed and identified by the first author and Melaku Wondafrash, an expert from the National Herbarium (ETH), through visual comparisons with authenticated plant specimens and using taxonomic keys in the volumes of Flora of Ethiopia and Eritrea [20–25]. Identifications were then authenticated by Prof. Sebsebe Demissew of Addis Ababa University, Ethiopia. Voucher specimens were deposited at the National Herbarium (ETH), Addis Ababa University. Selection of plant species was based on use reports of local informants and traditional herbalists from the study area for treatment of ailments caused by microbial agents. Ethnomedicinal use reports of the 15 medicinal plant species selected, parts used, and route of administration are summarized in Table 1.

**Table 1:** Ethnomedicinal use profile of tested medicinal plants, parts used, route of administration (ROA) and dried residue plant extract yield

No.	Botanical name [Family]	Local name	Part used	Ethnomedicinal use (Local name)	ROA	Yield (%)	Collected site	Voucher no.
1	*Apodytes dimidiata* E. Mey. ex Arn. [Icacinaceae]	Wendemu, Gefe	Bark	Cholera (Ye-dengiya-qar)	Oral	29	08°09.349′ N	At-85
							038°19.713′ E	
							2227 m a.s.l.	
2	*Asparagus africanus* Lam. [Asparagaceae]	Yefur ded	Leaf	Herpes zoster	Topical	21	08°01.370′ N	At-176
							038°21.219′ E	
							2031 m a.s.l.	
3	*Bersama abyssinica* Fresen. [Melianthaceae]	Hureta	Seed	Dandruff, wound, skin burn, scabies	Topical	33	08°15.799′ N	At-15
							037°46.261′ E	
							1793 m a.s.l.	
4	*Cucumis ficifolius* A. Rich. [Cucurbitaceae]	Hulgerecho, Yafer geranger, Yale tay, Adeni debaqula	Root	Abdominal pain, abdominal bloating, jaundice (Qoya), anthrax (Shem-itere), indigestion	Oral	17	08°02.189′ N	At-157
							038°31.220′ E	
							1826 m a.s.l.	
5	*Gladiolous abyssinicus* (Brongn. ex Lemaire) Goldblatt & de Vos [Iridaceae]	Enzerziye	Bulb	Toothache	Topical	41	08°08.024′ ′N	At-132
							037°55.70′ E	
							2065 m a.s.l.	
6	*Guizotia schimperi* Sch. Bip. ex Walp. [Asteraceae]	Mocho	Leaf	Wound, dandruff	Topical	32	08°01.370′ N	At-45
							038°21.219′ E	
							2031 m a.s.l.	
7	*Lippia adoensis* Hochst. ex Walp. var. *adoensis* [Verbenaceae]	Kessie	Leaf	Toothache, abdominal pain, diarrhea, indigestion	Oral, Topical	22	08°02.189′ N	At-59
							038°31.220′ E	
							1826 m a.s.l.	
8	*Olinia rochetiana* A. Juss. [Oliniaceae]	Tife	Bark	Toothache	Topical	37	08°08.024′ N	At-93
							037°55.70′ E	
							2065 m a.s.l.	
9	*Pavonia urens* Cav. [Malvaceae]	Menatef	Leaf	Diarrhea, indigestion, excess vomiting	Oral	25	08°07.924′ N	At-191
							038°21.969′ E	
							2143 m a.s.l.	
10	*Premna schimperi* Engl. [Lamiaceae]	Teqoqi	Leaf	Toothache	Topical	31	08°08.380′ N	At-122
							038°20.445′ E	
							2143 m a.s.l.	
11	*Pittosporum viridiflorum* Sis [Pittosporaceae]	Hunbosho	Leaf	TB, pneumonia	Oral	34	07°43.752′ N	At-251
							038°06.954′ E	
							2002 m a.s.l.	
12	*Polygala sadebeckiana* Gurke [Polygalaceae]	Shime yeter chiza, Felfel, Qiteriye,	Root	Anthrax, toothache, indigestion	Oral, Topical	57	08°15.799′ N	At-112
							037°46.261′ E	
							1793 m a.s.l.	
13	*Sida rhombifolia* L. [Malvaceae]	Badefacha	Root	Abdominal pain, amoebiasis	Oral	15	08°01.370′ N	At-13
							038°21.219′ E	
							2031 m a.s.l.	
14	*Solanum incanum* L. [Solanaceae]	Embuay	Fruit	Dandruff, anthrax, tonsillitis, wound	Oral, Topical	45	08°08.024′ N	At-155
							037°55.70′ E	
							2065 m a.s.l.	
			Root	Abdominal pain	Oral	22		
15	*Thunbergia ruspolii* Lindau [Acanthaceae] (Endemic)	Yangacha qomet	Leaf	Abdominal pain, general malaise (Michi)	Oral	16	08°08.024′ N	At-124
							037°55.70′ E	
							2065 m a.s.l.	
			Root	Cholera, abdominal pain, hemorrhoids	Oral	33		

### Preparation of plant extracts

Plant materials were air-dried and ground into powder using an electric mill (GM100 Retsch, Germany). Each powdered sample species (15 g) was macerated with 450 ml of 80 % ethanol and placed on a shaker (200 rpm) (GFL3005, Germany) for 24 h. All procedures, stated above, were carried out at room temperature. Extracts were then filtered and concentrated using a rotary vacuum evaporator (R-200 Buchi, Switzerland) at 40 °C. Dried residues were dissolved in 100 % dimethyl sulfoxide (DMSO) to obtain a stock concentration of 51.2 mg/ml, which was kept at −80 °C until use. Dried residue yield figures (%) are shown in Table 1.

### Chemicals

Antibiotics ciprofloxacin (purity 99.5 %), oxacillin (purity ≥ 81.5 %), tetracycline (purity ≥ 88 %) and tioconazole (purity 97 %), were purchased from Sigma-Aldrich (Prague, Czech Republic). Potency of the powder was incorporated in the formula for preparation of stock solutions according to EUCAST [26]. DMSO (Penta, Czech Republic), ethanol (Sigma-Aldrich, Czech Republic), and distilled water were used as solvents. Cation-adjusted Mueller-Hinton broth (MHB) (Oxoid, United Kingdom) equilibrated for testing with Tris-buffered saline (Sigma-Aldrich, Czech Republic) was used as a bacterial culture media.

### Microorganisms

In this study, 20 bacterial strains and one yeast were tested. The following American Type Culture Collection (ATCC) standard strains were purchased from Oxoid (United Kingdom) for analysis: *Candida albicans* ATCC 10231, *Enterococcus faecalis* ATCC 29212, *Escherichia coli* ATCC 25922, *Pseudomonas aeruginosa* ATCC 27853, *Staphylococcus aureus* (ATCC 25923, ATCC 29213, ATCC 33591, ATCC 33592, ATCC 43300, ATCC BAA 976), and *S. epidermidis* ATCC 12228. Ten clinical isolates of antibiotic-sensitive as well as antibiotic-resistant *S. aureus* strains (SA1, SA2, SA3, SA4, SA5, SA6, SA7, SA8, SA9, SA10) were provided by University Hospital in Motol (Prague, Czech Republic). Microorganism cultures were stored in MHB at 4 °C until use. Prior to antimicrobial tests, microorganisms were re-cultured at 37 °C for 24 h (48 h for *C. albicans*).

### Assessment of minimum inhibitory concentrations (MICs)

MICs were determined by the broth microdilution method using 96-well microplates modified according to previous recommendations for effective assessment of the anti-infective potential of natural products [27, 28]. An aliquot of 100 μl of two-fold serial dilutions of each extract was prepared in MBH, equilibrated with Tris-buffered saline, in concentrations ranging 4–512 μg/ml. For inoculum standardization, the turbidity of the bacterial suspension was adjusted to 0.5 McFarland standard (1.5 × 10^8^ CFU/ml) using Densi-La-Meter II (Lachema, Czech Republic) spectrophotometric device. This bacterial suspension was inoculated into each well, and plates were incubated at 37 °C for 24 h (48 h for *C. albicans*). Microorganism growth was measured as turbidity recorded at 405 nm using the Multiscan Ascent Microplate Reader (Thermo Fisher Scientific, Waltham, MA). The MIC was calculated as the lowest concentration that showed ≥ 80 % reduction of microbial growth compared to extract-free growth control. Antibiotics ciprofloxacin, oxacillin, teteracycline and tioconazole were used as positive controls. Oxacillin and teteracycline were used as markers for methicillin and tetracycline resistance, respectively. Solvents used did not inhibit bacterial growth at concentrations tested. We used *S. aureus* ATCC 29213 as a quality-control strain for antibiotic susceptibility. Results reported in this study are expressed as the mode of MICs obtained from three independent experiments that were assayed in triplicate.

## Results

Extracts from leaves of four species (*Asparagus africanus, Guizotia schimperi*, *Lippia adoensis* var. *adoensis*, *Premna schimperi*) showed activity against some of the tested microorganisms (Table 2). The extracts were active against *C. albicans, E. faecalis* and *S. aureus* at a concentration between 128 and 512 μg/ml. *Guizotia schimperi, L. adoensis* var. *adoensis* and *P. schimperi* showed activity against *E. faecalis* and *S. aureus* (MIC range from 128 to 512 μg/ml), whereas *A. africanus* inhibited growth of *E. faecalis* (MIC = 512 μg/ml). *Candida albicans* was susceptible to *G. schimperi* and *L. adoensis* var*. adoensis* at highest concentrations only (MIC = 512 μg/ml). Gram-negative bacteria (*E. coli* and *P. aeruguinosa*) were resistant to all ethanol extracts tested in this study.

**Table 2:** Minimum inhibitory concentration (MIC) of the medicinal plant species extracts

	MIC (μg/ml)				
	*Enterococcus faecalis* ATCC 29212	*Staphylococcus aureus* ATCC 25923	*Escherichia coli* ATCC 25922	*Pseudomonas aeruguinosa* ATCC 27853	*Candida albicans* ATCC 10231
*Apodytes dimidiata*	–	–	–	–	–
*Asparagus africanus*	512	–	–	–	–
*Bersama abyssinica*	–	–	–	–	–
*Cucumis ficifolius*	–	–	–	–	–
*Gladiolous abyssinicus*	–	–	–	–	–
*Guizotia schimperi*	128	128	–	–	512
*Lippia adoensis* var. *adoensis*	256	256	–	–	512
*Olinia rochetiana*	–	–	–	–	–
*Pavonia urens*	–	–	–	–	–
*Premna schimperi*	512	512		–	–
*Pittosporum viridiflorum*	–	–	–	–	–
*Polygala sadebeckiana*	–	–	–	–	–
*Sida rhombifolia*	–	–	–	–	–
*Solanum incanum*	–	–	–	–	–
*Thunbergia ruspolii*	–	–	–	–	–
*ATB*	0.5^a^	0.5^a^	0.015^a^	0.125^a^	0.5^b^

*ATB* Antibiotics used as positive control

^a^Ciprofloxacin

^b^Tioconazole

“–” No inhibition (MIC > 512 μg/ml)

The ethanol extract of *G. schimperi*, which showed strong activity against *E. faecalis* and *S. aureusas* as compared with other plant extracts, was subjected to further antibacterial analysis against 16 standard and clinical isolates of staphylococcal strains. The clinical isolates were resistant to either oxacillin (MIC ≥ 4 μg/ml) or tetracycline (MIC ≥ 16 μg/ml). Three isolates (SA2, SA3 and SA9) were resistant to both antibiotics, and can be considered as multidrug-resistant strains. Strong antibacterial activity was observed for *G. schimperi* extract against all strains tested at concentrations of 128–256 μg/ml (Table 3). Moreover, this extract showed higher antibacterial activity for tests against *S. aureus* ATCC 33591, ATCC 33592, SA3 and SA5 strains (128–256 μg/ml) than oxacillin (512 μg/ml). The same MIC values (128 μg/ml) were obtained for *G. schimperi* extract as for tetracycline in the test against ATCC 33592.

**Table 3:** *In vitro* anti-staphylococcal activity of *Guizotia schimperi* extracts and of antibiotics oxacillin and tetracycline

	MIC (μg/ml)		
Standard strains	Oxacillin	Tetracycline	Extract
ATCC 12228	0.5	64	128
ATCC 29213	0.5	0.5	128
ATCC 33591	512	64	128
ATCC 33592	512	128	128
ATCC 43300	16	0.25	256
ATCC BAA 976	16	0.25	128
Clinical isolates			
SA1	16	0.25	128
SA2	64	16	256
SA3	512	32	128
SA4	16	0.25	128
SA5	256	0.25	256
SA6	0.5	8	256
SA7	1	16	128
SA8	16	0.125	128
SA9	128	64	256
SA10	0.5	0.25	128

SA1-SA10 = resistant if MIC ≥ 4 μg/ml for oxacillin, ≥ 16 μg/ml for tetracycline [47]

*MIC* Minimum inhibitory concentration, *ATCC* American type culture collection, *SA Staphylococcus aureus*

## Discussion

The extracts tested in the present study revealed the potential of traditional medicinal plants in searching for novel pharmaceuticals. We explored 15 plants used in the Gurage and Silti Zones of Ethiopia. Gram-positive bacteria were more sensitive to the medicinal plant extracts tested than Gram-negative bacteria, consistent to previous findings [29, 30]. The *G. schimperi* extract inhibited all standard and clinical isolates of *S. aureus* tested. The latter bacterium has been stated as one of the leading causes of human infections, causing significant nosocomial illness, generally via hospital-acquired infections [31]. It occurs commonly in Ethiopia, and shows high levels of resistance to commonly-used antibiotics [2]. In this study, antibacterial activity was most pronounced against ATCC 33591, ATCC 33592, SA3 and SA5, with *G. schimperi* exhibiting higher activity (MIC ≤ 256 μg/ml) than oxacillin (MIC = 512 μg/ml). Togan et al. [32] described possible differences in susceptibility patterns between standard and clinical strains, in which clinical strains may represent current isolates responsible for clinical disease and spread of resistance.

To the best of our knowledge, no studies related to antimicrobial activity of *G. schimperi* (synonym of *G. scabra* subsp. *schimperi*) have been published previously. This annual weed named “Mocho” by the local people is very close taxonomically to *G. abyssinica* and *G. scabra* [24]. It is most likely the wild progenitor of *G. abyssinica*, cultivated for its edible seeds and known for its medicinal uses [33]. Chemical analysis of essential oils from *G. scabra* leaves collected from Rwanda has characterized germacrene-D, limonene and diterpenes as the principal constituents. These components have been shown to exhibit several medicinal properties [34, 35]. From a chemotaxonomic point of view, different plant species in a genus often share similar chemical components [36]. In view of these facts, the inhibition exhibited by *G. schimperi* against standard and clinical isolates in particularly at comparable concentration to standard antibiotics is very promising for phytomedicine development, so phytochemical investigation of *G. schimperi* leaves is needed to identify their antimicrobial active constituents.

Antimicrobial analysis of *L. adoensis* var. *adoensis* extract showed activity against *C. albicans, E. faecalis* and *S. aureus*. However, no activity against *E. coli* or *P. aeruginosa*. In other studies, petroleum ether, chloroform, acetone and methanol extracts of *L. adoensis* var. *adoensis* showed significant activity against *E. coli* and *P. aeruginosa* [17] but were inactive against *C. albicans* [16]. Wasihun et al. [17] reported presence of secondary metabolites of *L. adoensis* responsible for its antimicrobial activity. The latter authors further showed that, non-polar fractions have relatively better antimicrobial activity compared to polar fractions. Motamedi et al. [37] report that solubility of active principles in plant materials varies according to extraction solvent used, which may relate to differences in antimicrobial effect of plant extracts [38]. Hence, the extraction solvents used in this study could have caused variation in the antimicrobial activity results.

*Asparagus africanus* extracts showed activity against *E. faecalis. Asparagus* spp. contain steroidal saponins as major bioactive constituents besides others including, such as flavonoids, resins and tannins [39]. Our results could reflect the bioactive constituents mentioned above. Madikizela et al. [40] applied the broth microdilution method and reported *A. africanus* ethanol extract as inactive against *S. aureus*, which complements our results. We found that *P. schimperi* inhibited growth of *E. faecalis* and *S. aureus*. Habtemariam et al. [19]reported a novel diterpene in leaves as active against *S. aureus*, which might explain the antibacterial activity of *P. schimperi* in our study.

Extracts of *Solanum incanum* fruit (methanol, hexane and chloroform) tested by disc diffusion and broth dilution techniques showed no activity against *E. coli, S. aureus* and *P. aeruginosa* [41], matching our findings. Alamri and Moustafa [42] applied agar well diffusion to test ethanol extracts of *S. incanum* fruit, and found it very active against *S. aureus*, with less activity against *P. aeruginosa* and *E. coli*. In a similar study, phenolic compounds were isolated from *S. incanum* fruits, which could be responsible for inhibition of *S. aureus* [42]. Concentration of active principles in plants may vary with climate and across geography [15]. Moreover, different methodologies may contribute to differences in antibacterial activity, particularly in the case of our *S. incanum* fruit extracts.

In the present study, some of the plant species tested on antimicrobial activity showed no inhibition within the applied concentration ranges. Known medicinal plants, such as *Apodytes dimidiata* (bark), *Olinia rochetiana* (bark) and *Polygala sadebeckiana* (root), have been claimed to be medicinally useful by local communities of the study area and in previous scientific studies [29, 30, 43]. The methanol extracts of *O. rochetiana* bark exhibited antiviral activity against measles virus [43], whereas the anticancer agent, camptothecine, was isolated from the bark of *A. dimidiata* [44]. For *P. sadebeckiana*, apart from the ethnomedicinal uses reported by Hailemariam et al. [45] in Ethiopia (the root being used to cure liver disease, abdominal distention and snake bite), no information was found on its medicinal use and antimicrobial effects. It is further also possible that ethanol extract, plants that showed no inhibition, is only active at higher concentrations than the starting concentration (512 μg/ml) used in our study. In general, the disparities between our findings and others may result from differences in chemical composition of extracts, effects of secondary metabolites including antiviral properties [46], geographic variation in antimicrobial properties, or methodological considerations. Scientific testing of medicinal properties thus need to consider these diverse factors, such that application of different testing methods and extraction solvents is important. Regarding species that resulted inactive in this study, despite strong claims of medicinal value, further analyses are needed before more conclusions can be drawn.

## Conclusions

The present study revealed the potential of some traditional medicinal plants to be used as sources of antimicrobials. The usefulness of these plants, in particular of *G. schimperi*, should be confirmed through further phytochemical and toxicity analyses.
